# Left Prefrontal Cortex Supports the Recognition of Meaningful Patterns in Ambiguous Stimuli

**DOI:** 10.3389/fnins.2020.00152

**Published:** 2020-02-21

**Authors:** Grégory Bartel, Martin Marko, Imani Rameses, Claus Lamm, Igor Riečanský

**Affiliations:** ^1^Social, Cognitive and Affective Neuroscience Unit, Department of Cognition, Emotion, and Methods in Psychology, Faculty of Psychology, University of Vienna, Vienna, Austria; ^2^Department of Behavioural Neuroscience, Institute of Normal and Pathological Physiology, Centre of Experimental Medicine, Slovak Academy of Sciences, Bratislava, Slovakia; ^3^Department of Applied Informatics, Faculty of Mathematics, Physics and Informatics, Comenius University in Bratislava, Bratislava, Slovakia; ^4^Vienna Cognitive Science Hub, University of Vienna, Vienna, Austria

**Keywords:** tDCS, prefrontal cortex, visual perception, object recognition, Rorschach test, semantic memory, divergent thinking, convergent thinking

## Abstract

Processing of ambiguous visual stimuli has been associated with an increased activation of the left lateral prefrontal cortex (PFC) in neuroimaging studies. Nevertheless, the functional role of prefrontal activity in this process is not fully understood. In this experiment we asked participants to evaluate ambiguous inkblots from the Rorschach test, while stimulating the left lateral PFC using excitatory anodal transcranial direct current stimulation (tDCS). In addition, visual insight ability was assessed as a control measure requiring visual and conceptual restructuring and convergent thinking rather than divergent idea generation employed to interpret the equivocal Rorschach inkblots. Using a randomized double-blind design, we demonstrated that anodal tDCS increased the number of meaningful patterns recognized in the inkblots but had no significant effect on visual insight. These findings support the role of left lateral PFC in the processing of ambiguous visual information and object recognition. More generally, we discuss that the PFC may be involved in the mechanisms supporting the activation of stored visual and semantic representations in order to compensate for less informative bottom-up inputs and thus facilitate flexible cognition and idea generation.

## Introduction

Seeing is not merely a bottom-up process, but also uses the constructive qualities of our perceptual system, which are essential to navigate in a world full of ambiguity and incompleteness (Gregory, 1970, 1990). An important aspect of adaptive behavior entails a dynamic interpretation of elements embedded in natural scenes, which are ambiguous due to various forms of noise (i.e., degraded visibility, shading, and occlusion) or intrinsic complexity. By virtue of this ambiguity, natural visual scenes frequently produce perceptions of non-existent entities, reflecting approximate or even erroneous matching between sensory inputs and internal representations, commonly referred to as illusory perception, pareidolia, or projection (Voss et al., 2012; Liu et al., 2014). Such phenomena are based on top-down processes using implicit knowledge and semantics, which provide us with the ability to interpret specific forms as Gestalts (Gregory, 2009), thus making sense of patterns perceived in the visual field.

The processing of ambiguous and degraded visual patterns engages areas of the prefrontal and parietal cortex (Bar et al., 2006; Cho-Hisamoto et al., 2015), indicating an increased reliance on top-down mechanisms or executive control during recognition of ambiguous objects. More specifically, it has been suggested that coarse representations of incoming stimuli are carried rapidly from early visual areas to the prefrontal cortex (PFC) in order to compute the prediction about the most likely interpretation of the input (Summerfield et al., 2006). This top-down prediction precedes and augments the bottom-up processing in the temporal-occipital visual pathway, supporting rapid recognition of objects (Bar, 2004; Bar et al., 2006). Ambiguous or distorted visual inputs may compromise the meticulous bottom-up analysis performed in the temporal-occipital cortices thus increasing the reliance on prefrontally mediated top-down processes.

Transforming sensory inputs into meaningful patterns is further supported by the semantic system, as indicated by the activation of the left-hemispheric language network during object recognition (Gabrieli et al., 1998; Chouinard et al., 2009; Ralph et al., 2017). Notably, the processing of ill-structured or noisy visual patterns further increases the activation of this network, particularly in the left inferior frontal cortex (Liu et al., 2010, 2014; Luciani et al., 2014). Interestingly, such activity increase in the PFC has also been reported during semantic memory retrieval, especially when the stimuli provide insufficient retrieval cues (Badre and Wagner, 2002) or when multiple instances have to be retrieved in rapid succession (Ghanavati et al., 2019). Further, PFC is involved in facilitation of semantic retrieval to support the bottom-up processing during ambiguous object recognition (Eger et al., 2007). This suggests that the left lateral PFC may employ a more extensive semantic analysis to compensate for less informative bottom-up inputs.

Conclusively, numerous neuroimaging studies suggest that left lateral PFC is involved in the processing of ambiguous or distorted visual inputs. Nevertheless, the contribution of the PFC and its functional role in this cognitive process remains unclear. For this purpose, we targeted the left lateral PFC with transcranial direct current stimulation (tDCS), previously shown to modulate neural excitability of the PFC and its functional connectivity within the task-related brain networks (Keeser et al., 2011; Pisoni et al., 2018). Ambiguous object processing was assessed as response fluency in the Rorschach inkblot test (ROR; Rorschach, 1921). ROR involves the search and detection of visual features, mental imagery, retrieval of knowledge, and (paced) formulation of meaningful interpretations of ambiguous visual stimuli (inkblots). In addition to the temporal-occipital visual pathways, ROR and similar tasks recruit the PFC (Aziz-Zadeh et al., 2013; Luciani et al., 2014; Giromini et al., 2017), suggesting that top-down information mediates the interpretation of ambiguous visual stimuli. Based on these findings, we predicted that excitatory anodal tDCS over the left lateral PFC would facilitate the prefrontal computations that augment ambiguous object processing, which would result in an increased number of patterns recognized in the ROR task. Furthermore, we also assessed visual insight ability as a comparison measure in order to address the specificity of the expected effect. In contrast to ROR, insight problems do not involve ambiguous visual shapes (i.e., participants instantaneously recognize distinct visual elements that constitute a problem) but their solutions require a conceptual reinterpretation, through meaningful rearrangement of the elements of the problem (Ash and Wiley, 2006; Marko et al., 2019b). This specific cognitive process has been associated with activation within temporal-occipital regions rather than the PFC (Chi and Snyder, 2011, 2012; Kounios and Beeman, 2014). In addition, insight problems put explicit constraints on the appropriateness of answers (i.e., convergent thinking), whereas ROR engages divergent thinking, which has been coupled with left PFC activation (Wu et al., 2015; Pidgeon et al., 2016). Therefore, we did not expect to see a significant influence of the tDCS on both the ROR and the insight performance, as it would indicate that neurostimulation modulates general visual cognitive processing, including visual reasoning and (convergent) problem solving.

## Materials and Methods

### Participants and Study Design

Forty healthy volunteers (21 males and 19 females; mean age 25.0 ± 4.2 years) participated in the study. All participants were right-handed, as assessed by the Edinburgh Handedness Inventory (Oldfield, 1971), with no history of psychiatric or neurological disorders, or current use of medication. The participants had normal or corrected-to-normal vision and intact color discrimination. Written informed consent was obtained from all participants. The study was approved by the appropriate ethics committee (Ethics Committee of the Institute of Normal and Pathological Physiology CEM SAS) and conducted in accordance with the Declaration of Helsinki. The participants received either academic credits or financial compensation after completing the session. The study had a randomized, double-blind, between-subjects design: the participants were randomly assigned to receive either active or sham stimulation. The active and the sham group did not significantly differ in the average age, *t*(37) = 0.444, *p* = 0.659, *d* = 0.14, sex, χ*2*(1, *N* = 40) = 0.902, *p* = 0.342, and positive and negative affect, which was assessed both before and after tDCS, *t*(36) < 0.846, *p* > 0.403, *d* < 0.28.

### Procedure

**FIGURE 1:**
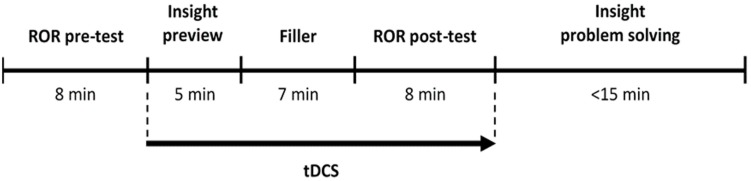
Experimental procedure. The arrow indicates the period during which (sham or active) tDCS was applied.

The experimental session started with a short interview, in which participants were informed about the study and the neurostimulation protocol. Thereafter, stimulation electrodes were attached and participants were comfortably seated in a quiet room approximately 50 cm away from a screen with a refresh rate of 60 Hz (color calibration was kept identical across all sessions). Participants then completed the Positive and Negative Affect Schedule (PANAS; Thompson, 2007), which was introduced to examine possible group differences in participants’ emotional state. After the questionnaire administration, the experimenters explained the cognitive tasks to the participants, followed by a short period of practice. The main experiment (Figure 1) began with a baseline measurement of ROR (ROR pre-test), which was administered prior to the onset of the neurostimulation. Immediately after the stimulation started, participants were presented with a set of six visual insight problems (for 5 min, without the possibility to respond) and then watched a cartoon movie as a filler (for 7 min). After the movie was stopped, the participants completed a second ROR administration (ROR post-test), which lasted until the end of the stimulation period (8 min). Immediately afterward, the set of insight problems was reintroduced and participants were asked to solve the problems and describe the solutions (15 min). Finally, the participants’ affective state was re-assessed using PANAS, the participants were debriefed, and asked to guess whether they received sham or active stimulation. Overall, each session took approximately 70 min.

### ROR Response Fluency

Response fluency was assessed using all 10 inkblot tables from the Rorschach test (Rorschach, 1921). The average response rate for each table was calculated using a normative sample including 146 Rorschach interviews (Sultan et al., 2004). The tables were then divided into two sets of equal size and average response rate (average response rate ± SD, set 1: 2.3 ± 0.3, including three colored inkblots; set 2: 2.3 ± 0.7, including two colored inkblots). The two sets were counterbalanced across two assessment blocks (pre-test and post-test). In both assessment blocks, the five inkblot tables were sequentially presented on a computer screen with white background in random order for 90 s (the duration was chosen based on preliminary tests). The size of the inkblots was 23 × 17 cm. In each trial, participants were asked to think of and name as many things as they could see in the ambiguous inkblot following the question “What do you see in the picture?”. Each response was indicated by a keypress using computer keyboard, after which the participants typed in the full content of the response (e.g., “a big black giant”). In order to exclude nonsense responses (i.e., non-words or typos), participants’ responses were evaluated by the experimenters using a blinded protocol, and two responses were excluded from the analyses. Since ROR was used as a performance test in the present study, other qualitative aspects putatively reflecting personality traits and/or clinical markers were not assessed. The performance score reflected the total number of valid responses evoked by the five tables within each assessment block. The internal consistency of the performance scores was high (Cronbach’s α > 0.85).

### Insight Problems

Insight problem solving was assessed using six visual insight problems; the set included the “10-coin triangle problem” (De Bono, 1969), “9-dot problem” (Kershaw and Ohlsson, 2004), two matchstick arithmetic problems (Knoblich et al., 1999), the “pigs in a pen,” and “the inverted pyramid” problems (Schooler et al., 1993), presented in fixed order. First, participants were given the opportunity to simply view each problem and think about a possible solution for 50 s, without providing any response at this stage. Then, after the stimulation had finished, the problems were presented again for 180 s each; thus each problem was displayed for 230 s in total, which was similar to previous studies (Schooler et al., 1993; Knoblich et al., 1999; Kershaw and Ohlsson, 2004; Weller et al., 2011). This time, participants were asked to write down the correct solution to the problems as quickly as possible. The insight performance was therefore evaluated using the insight score (the number of correctly solved problems) and average solving time (insight reaction time, RT). One matchstick problem was excluded from further analyses due to low item-total correlation (polychoric correlation coefficient, *r* = 0.054). The “9-dot problem” was excluded as well, since several participants reported previous knowledge of its solution. The internal consistency of the remaining four insight problems was acceptable, as indicated by polychoric Cronbach’s alpha (α = 0.79). The insight RTs were also consistent (α = 0.70).

### tDCS

**FIGURE 2:**
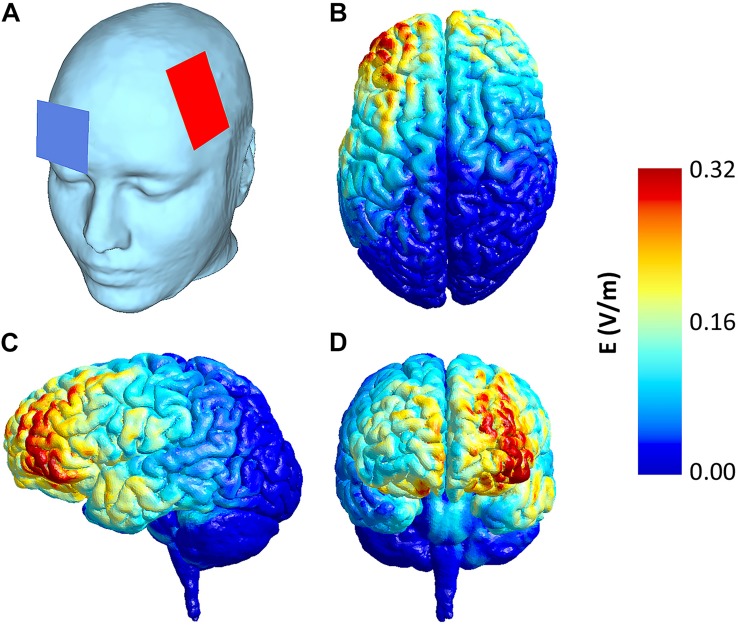
Electrode placement and simulated electric field for the tDCS montage. Anode was centered in between F3-F5 and cathode over Fp2 of the 10-10 international system of EEG electrode placement **(A)**. The estimated electric field intensity induced in the brain is shown in dorsal **(B)**, left lateral **(C)**, and frontal **(D)** view, based on a biologically plausible computational forward model of the current flow for this montage (for details see section “Materials and Methods”).

Stimulation was delivered by a certified battery-driven, constant current source (DC-STIMULATOR PLUS, NeuroConn, Ilmenau, Germany) and a pair of conductive rubber electrodes (7 × 5 cm). The electrodes were attached on the scalp using electrode paste (Ten20, Weaver and Co., Aurora, CO, United States) and kept firm by an EEG cap and elastic bands. The anode was placed over the left PFC, centered between F3 and F5 of the 10-10 EEG international system of electrode placement. The cathode was located over the right supraorbital region (Fp2). This montage was determined using biologically plausible computational forward models of brain current flow (Bikson et al., 2012) in SimNIBS software (version 3, Thielscher et al., 2015) and based on previous research targeting left PFC. The models were estimated using a high-quality head model (derived from MR images) provided by the software and the corresponding conductivities of the head and brain tissue. Using these parameters and electrode sites, the electric fields in the brain were estimated and subsequently visualized using Gmsh (a three-dimensional finite element mesh generator; Geuzaine and Remacle, 2009). The model for our tDCS montage indicated largest polarizations within the left dorsal and ventral lateral PFC (see Figure 2 for more details). The active stimulation was set to 1.5 mA (0.042 mA/cm^2^) and was delivered for 20 min, including 30 s of ramping up and down. The active stimulation was stopped after the second block of ROR assessment had been completed. In the sham condition, the stimulation included the initial ramp-up to evoke potential sensations as in the active condition. However, the current ramped down after 40 s (default device setting). Participants’ estimations on whether they received sham or active condition were not statistically different between the groups, χ*^2^*(1, *N* = 40) = 2.057, *p* = 0.342, indicating that the blinding protocol was effective.

### Data Processing and Analysis

The data were processed in R studio (RStudio Team, 2018) using R language and SPSS 25 (IBM corp.). The distributions of the ROR performance scores (i.e., total number of responses produced by each individual) were explored using boxplots. The analysis indicated three outlying values in the ROR data (1.5 interquartile range criterion), which were therefore winsorized using 5% trimming (two-tailed) prior to statistical analyses. The effects of tDCS on ROR response fluency was evaluated using an ANCOVA model (tDCS was used as a fixed binary between-subjects factor and pre-test performance scores were used as a continuous covariate). Group differences in insight performance were assessed using *t*-tests. Effect size was estimated using η_*p*_^2^ and Cohen’s *d*.

## Results

**FIGURE 3:**
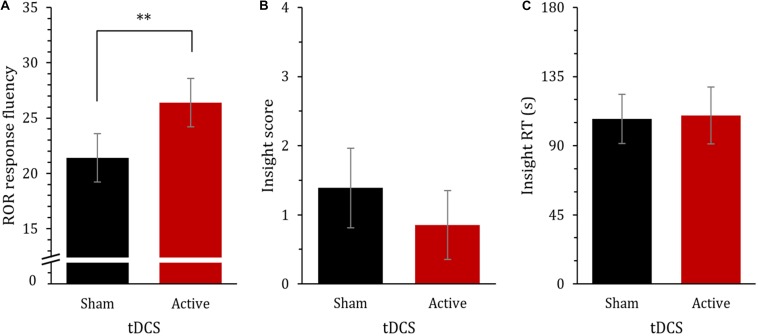
Performance in the cognitive measures by tDCS group for **(A)** ROR response fluency: the average number of responses delivered in the post-test when controlling for the pre-test, **(B)** insight score: the average number of correctly solved insight problems (max = 4 problems), and **(C)** insight RT: the average time required to solve the problems (max = 180 s). Error bars depict 95% CIs. ** *p* < 0.01.

A one-way ANCOVA was conducted to evaluate the effect of tDCS (sham vs. active stimulation) on ROR response fluency in the post-test whilst controlling for the pre-test performance. The analysis showed that the number of responses in the active group (*M* = 26.4, *SE* = 1.19) was higher than in the sham group (*M* = 21.4, *SE* = 1.19), which was statistically significant, *F*(1,37) = 10.27, *p* = 0.003, η_*p*_^2^ = 0.217 (Figure 3A). On average, active tDCS improved the performance by approximately five responses, 95% CI [1.82, 8.09]. The pre-test performance covariate was highly significant, *F*(1,37) = 2695.3, *p* < 0.001, η_*p*_^2^ = 0.754 (zero-order correlation between the pre-test and the post-test performance were comparable between the tDCS conditions: Pearson *r* = 0.89 and *r* = 0.83, for the sham and the active stimulation, respectively). Also, in order to verify that the observed effect of tDCS on ROR response fluency was not contingent on whether the inkblots were monochromatic or colored, we computed a repeated measures ANOVA including *tDCS* as a between-subject factor and *Block* (pre-test, post-test) and *Color* (monochromatic, colored) as two within-subject factors. The analysis showed no statistically significant interaction including *Color* factor (*F* < 0.143, *p* > 0.239).

The average solving accuracies of the four insight problems were rather low, ranging from 15 to 38%, and the average response times ranged from 87 s to 121 s (two participants were excluded from the analysis due to prior knowledge of the problems). The effects of tDCS on the insight performance was evaluated via two separate independent sample *t*-tests. The *t*-test for insight score did not show a statistically significant difference between the sham (*M* = 1.4, *SE* = 0.29) and the active tDCS group (*M* = 0.85, *SE* = 0.25), *t*(36) = 1.40, *p* = 0.171, *d* = 0.45 (Figure 3B). On average, the active group scored 0.54 points less than the sham group, 95% CI [−0.24, 1.32]. Similarly, the *t*-test for insight RT revealed no statistically significant difference between the groups, *t*(35) = 0.181, *p* = 0.858, *d* = 0.06 (Figure 3C). The average solving time was longer in the active (*M* = 109.8, *SE* = 9.7) than in the sham group (*M* = 107.4, *SE* = 8.7) by approximately 2.4 s, 95% CI [−28.8, 24.1]. The correlation between insight score and insight RT was not statistically significant, *r* = −0.175, *p* = 0.448 (Pearson correlation, two-tailed). The insight measures were not significantly associated with the ROR change score (post-test minus pre-test) in any tDCS group, *r* < 0.265, *p* > 0.181 (Pearson correlation, two-tailed).

## Discussion

We used anodal tDCS over the left lateral PFC to modulate ambiguous visual object processing assessed by using the Rorschach test (ROR). Our prediction was that the excitatory stimulation would facilitate the left prefrontal involvement in the processing of ambiguous inkblots and thus improve the number of meaningful patterns recognized in the ROR inkblots, i.e., divergent idea generation. On the other hand, we did not expect such an effect on the insight ability since solving insight problems employs convergent visual and conceptual restructuring, which had been previously associated with temporal-occipital areas. In line with our prediction, the participants undergoing active stimulation, compared with those receiving sham stimulation, showed increased response fluency in the ROR test, as measured by the number of responses per inkblot, but no effect on performance in visual insight tasks. These findings provide new evidence for the role of the PFC in the processing of ambiguous visual stimuli, suggesting that the PFC may support a top-down mechanism that aids object recognition when bottom-up inputs are less informative.

According to the predictive coding theory of perception, the brain constantly predicts the content of future sensory experience and compares the incoming sensory information against internally generated representations (Summerfield et al., 2006). If there is a sufficient match between the two, the brain interprets the stimulus in accordance with the generated internal representations. For visual perception, it has been suggested that coarse representations of the stimuli are transferred rapidly from early visual areas to the PFC, where an internal model that interprets the sensory input is computed (Bar, 2004; Bar et al., 2006). Although the predictions about physical and semantic content of visual stimuli are only approximate and prone to errors, they effectively facilitate processing and recognition of complex visual patterns. When a visual stimulus is unambiguous, the bottom-up processing implemented within the temporal-occipital brain circuits may be sufficient for rapid object recognition. On the other hand, however, if the input is ambiguous or degraded, the PFC may provide top-down estimations to compensate for less informative sensory inputs and thus facilitating the recognition of objects (Cho-Hisamoto et al., 2015). Interestingly, a very similar interaction between the prefrontal-temporal (top-down) and temporal-occipital (bottom-up) networks has been observed during mental projection (Luciani et al., 2014), i.e., subjective attribution of meaning to ill-structured stimuli or situations, suggesting that predictive coding may underpin this phenomenon. In summary, the observed improvement in ROR response fluency by anodal tDCS may stem from an increased excitability of the PFC, or the prefrontal-temporal brain network, that generates likely estimations of ambiguous stimuli, which in turn enhances the recognition of meaningful patterns. Notably, since the ability to solve visual insight problems does not include visual ambiguousness, their dependence on the prefrontal predictions may be limited, rendering the effects of tDCS insignificant.

From another viewpoint, ROR is also regarded as measure of divergent thinking that reflects creative potential of human individuals (Gregory, 2000; Aziz-Zadeh et al., 2013). Divergent thinking tasks require serial generation and evaluation of multiple unique ideas for a single stimulus (Guilford, 1967; Silvia et al., 2013). Several studies linked divergent thinking with the left lateral PFC (Vartanian and Goel, 2007; Fink et al., 2009; Colombo et al., 2015; Zmigrod et al., 2015). In particular, Aziz-Zadeh et al. (2013) reported that a broader language network in the left hemisphere, including lateral PFC, was engaged during divergent thinking even if visual (i.e., non-verbal) stimuli were used, suggesting that the left prefrontal computations support diverging ideational production across multiple sensory modalities and domains. This is consistent with the absent effect of anodal tDCS on the insight ability, as solving visual insight problems engages convergent mode of thinking that requires singling out a response from several possible alternatives in an analytical, deductive manner as well as strict evaluation of potential solution candidates. This form of insightful thinking and problem solving has been primarily associated with neural activity in the temporal-occipital cortical regions (see Chi and Snyder, 2011, 2012; Kounios and Beeman, 2014; Salvi et al., 2020). A positive influence of an inhibitory cathodal tDCS of the left lateral PFC on insight problem solving reported by Luft et al. (2017) indicates that this region may put constraints on insightful problem solving via top-down prediction bias. Yet, more research is needed to assess the role of left lateral PFC in convergent vs. divergent thinking more specifically.

The electric field model showed that our stimulation also reached the left inferior PFC. Evidence indicates that this area plays a pivotal role in semantic cognition (Ralph et al., 2017), especially in semantic retrieval and verbal fluency (Badre and Wagner, 2002; Costafreda et al., 2006; Fedorenko and Thompson-Schill, 2014; Marko et al., 2019a). In many aspects, ROR is similar to verbal fluency task, as both involve paced knowledge retrieval (Shao et al., 2014; Whiteside et al., 2016) and engage overlapping “semantic” circuits within the left hemisphere (Aziz-Zadeh et al., 2013; Luciani et al., 2014; Giromini et al., 2017). Moreover, several neurostimulation studies have demonstrated that single-session excitatory tDCS over the left lateral PFC increases the number of responses produced in verbal fluency tasks (Cattaneo et al., 2011; Price et al., 2015; Pisoni et al., 2018; Marko et al., 2019c), which parallels the findings of our study. Thus, since the response fluency in ROR and verbal fluency share several cognitive and neurophysiological processes, enhanced controlled semantic processing could play role in the improved ROR performance by tDCS. Further research evaluating the similarities between ROR fluency and verbal fluency is required to comprehensively address this account.

A wealth of data links the left lateral PFC with working memory, cognitive control and behavioral goal representations, as well as top-down control of information processing through selective attention (see e.g., Fuster, 2015). Several neurostimulation studies targeting left lateral PFC reported improved working memory capacity with anodal tDCS (Zaehle et al., 2011; Brunoni and Vanderhasselt, 2014; Marko et al., 2019c). However, such an effect would not explain our findings since ROR does not heavily exploit working memory capacity and does not require prolonged maintenance of memory contents. On the other hand, ROR may engage domain-general executive functions, such as inhibition of competing visual features or interpretations and switching of the attentional focus when scanning the inkblots (i.e., switching focus between global features and details or various parts of the inkblot). Future studies thus should investigate whether modulation of executive attention via tDCS (Imburgio and Orr, 2018) would also affect ROR performance.

Our study has several limitations to be considered. First, solving insight problems is inherently difficult and our results are consistent in this regard with the main body of previous research (Metcalfe, 1986; Schooler et al., 1993; Knoblich et al., 1999; Kershaw and Ohlsson, 2004; Weller et al., 2011). Despite an adequate reliability of the insight score, however, low solving rates could restrict the ability to sensitively detect a negative effect of the stimulation. Second, the insight performance was assessed in the post-test block only so that we cannot exclude pre-existing (random) group differences in this measure or a possibility that the administration of ROR interacted with the stimulation effects on the subsequent insight problem solving. Nevertheless, since previous research has shown that changes in neural excitability and connectivity induced by tDCS last for several tens of minutes after the stimulation (Stagg and Nitsche, 2011), it is not expected that the absent effects of the stimulation on the insight problem solving were due to timing issues. Third, our study did not assess physiological measures of the stimulation effects and combining neurostimulation with neuroimaging methods in next studies may provide more precise information about the induced functional changes in the brain. Finally, although a detailed evaluation of the qualitative aspects of ROR responses (such as elaboration and originality) was out of the scope of this study, it may provide interesting measures in future studies of human cognition.

## Conclusion

We have demonstrated that anodal electrical stimulation of the left PFC enhances response fluency in the Rorschach test. The study thus provides evidence for the involvement of the left lateral PFC in the processing of ambiguous information and reveals that left lateral PFC plays a role in the organization of visual object recognition. In light of previous research, we propose that the excitatory tDCS could enhance the prefrontal top-down computations that support object recognition when the bottom-up systems receive less informative or ambiguous sensory inputs. The observed improvements in ROR fluency could also result from enhanced controlled semantic cognition that enables more effective recognition of meaningful patterns in the ill-structured and ambiguous visual shapes. Our findings corroborate the growing evidence suggesting a pivotal role of the left lateral PFC in the control of cognitive processing across multiple domains (perceptual as well as semantic). In line with this role, we conclude that the excitatory stimulation of the left lateral PFC facilitates flexible integration of sensory input with internal representations. This function may support many cognitive skills ranging from the interpretation of ambiguous stimuli to productive mental ideation.

## Data Availability Statement

The datasets generated for this study are available on request to the corresponding author.

## Ethics Statement

The studies involving human participants were reviewed and approved by the Ethics Committee of the Institute of Normal and Pathological Physiology CEM SAS. The patients/participants provided their written informed consent to participate in this study.

## Author Contributions

GB, MM, IRa, and IRi designed the experiment and wrote the manuscript. GB and IRa collected the data. MM analyzed the data. All authors contributed to the interpretation of the results and critical discussion, and read and approved the submitted version of the manuscript.

## Conflict of Interest

The authors declare that the research was conducted in the absence of any commercial or financial relationships that could be construed as a potential conflict of interest.
